# Imidazole Derivative Cyclotriphosphazene-Based PLA/PEG Composite Films: Antibacterial Properties against *Escherichia coli* and *Staphylococcus aureus*

**DOI:** 10.1021/acsomega.5c06436

**Published:** 2025-08-28

**Authors:** Irem Demir, Gamze Seker, Arzu Aysan, Merve Dandan Doganci, Derya Davarcı, Erdinc Doganci

**Affiliations:** † Department of Chemistry, 52962 Gebze Technical University, 41400 Gebze, Kocaeli, Turkey; ‡ Department of Molecular Biology and Genetics, Gebze Technical University, 41400 Gebze, Kocaeli, Turkey; § Department of Chemistry and Chemical Processing Technologies, 52980 Kocaeli University, 41001 Kocaeli, Turkey

## Abstract

In this study, antimicrobial poly­(lactic acid)/poly­(ethylene glycol) (PLA/PEG) biofilms containing imidazole derivative cyclotriphosphazene compounds were prepared by using the solvent casting method. The mechanical, thermal, and biologically active properties of the obtained films were investigated. Biofilms were formed by adding the synthesized fully substituted methylimidazole and benzimidazole cyclotriphosphazene compounds (MCp and BCp) to the PLA/PEG mixture as additives at different rates. Tensile tests were performed on these films to examine their mechanical properties, including Young’s modulus, elongation at break, and yield strength. It was observed that the mechanical properties of the films were slightly negatively affected by the addition of additives to the PLA/PEG films. When thermal stability analyses were examined, it was determined that the addition of the additives increased slightly, although not significantly. Antimicrobial tests were performed using Gram-negative *Escherichia coli* ATCC 53323 and Gram-positive *Staphylococcus aureus* ATCC 29213 bacterial strains for MCp and BCp and PLA/PEG composite films MCp and BCp, and PLA/PEG composite films containing these compounds. It was observed that MCp was much more effective than BCp in inhibiting the growth of Gram-positive and Gram-negative bacteria. Additionally, the PLA/PEG/MCp and PLA/PEG/BCp films had antibiofilm activity.

## Graphical abstract

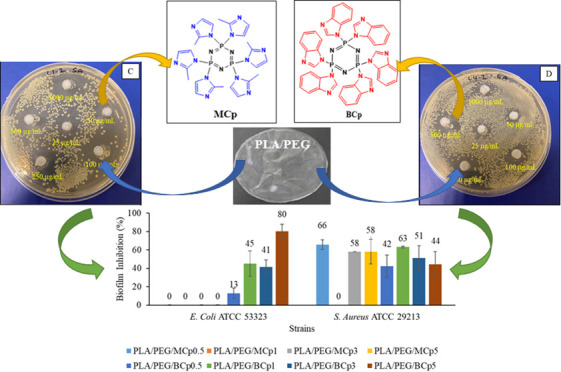

## 1 Introduction

Poly­(lactic acid) (PLA) and poly­(ethylene glycol) (PEG)-based biomaterials are frequently preferred in many different fields due to their renewable, biocompatible and biodegradable properties.^1^ PLA/PEG films are potential carriers for drug delivery in breast and lung cancer treatment,^2–4^ tissue engineering,^5^ and bioink^6^ applications. PLA and PEG-based biomaterials have recently been used in combination with various phytochemicals^7^ or synthetic^8^ bioactive agents to provide antibacterial and antibiofilm properties. Imidazole and its derivatives are heterocyclic compounds with a five-membered ring containing two nitrogen atoms and are well-known compounds that have certain properties of various medicinal agents. In recent years, imidazoles, including natural and synthetic imidazoles, have begun to play a role in the synthesis of many biologically active molecules. They exhibit a variety of biological activities, including antifungal, antibacterial, anti-inflammatory, analgesic, antidepressant, and anticancer.^9–16^ Antibacterial and antibiofilm effects of nitroimidazole derivatives coded as 8a–8o in related reference^17^ were evaluated on *E. coli* and *Staphylococcus aureus*. In particular, compound 8g showed a low MIC (1 μg/mL) and high antibacterial activity against methicillin-resistant *Staphylococcus aureus* (MRSA) and one methicillin-susceptible *S. aureus* (MSSA) isolates, while compounds 8i and 8m were more effective than metronidazole against carbapenem-resistant *E. coli* isolates. Compounds containing a piperazine group showed a stronger antibacterial effect, while compounds 8a, 8b, 8c, 8e, 8f, 8g, 8i, 8k, 8m, and 8n exhibited significant antibiofilm effects. Copolymers of vinyl imidazole (VI) and hydroxyethyl methacrylate (HEMA) in different molar ratios were synthesized. These copolymers were added to chitosan to prepare the films. The antibacterial performance of these films was observed to be that the blend film containing a high molar ratio of VI formed an inhibition zone for Gram-positive and Gram-negative bacteria. The findings demonstrated the films’ versatility in biological applications, including food packaging systems.^18^ Sugiyama and Bando synthesized *N*-methylimidazole-containing polyamides for sequence-specific DNA alkylation because of the biological activity of imidazoles.^19^ Drozd et al. synthesized water-soluble chitosan derivatives containing *N*-methylimidazole moieties via click chemistry. When methylimidazole is present in chitosan, the antibacterial activity of chitosan derivatives is four times more than that of the original chitosan for the microorganisms under study.^20^ Chitosan derivatives bearing imidazole rings, including the Schiff bases of chitosan, were designed and synthesized. Antibacterial properties were investigated, and results showed that quaternized chitosan is synergistic with imidazole groups and has better antioxidant, antifungal, and antibacterial properties than chitosan.^21^ Imidazole-loaded PLA–PEG films can be used in various biomedical and industrial fields due to their antibacterial and antibiofilm properties. These films have been reported to prevent bacterial infections and accelerate the healing of chronic wounds,^22^ dermatological treatments against skin infections,^23^ thanks to imidazole compounds. Therefore, it is essential to evaluate the potential of imidazole-loaded PLA–PEG films as biodegradable coatings for the control of resistant infections in long-term treatments and to reduce the risk of infection in medical implants, devices, or prosthesis applications. Imidazole loading can provide a significant advantage in the treatment of bacterial infections and the prevention of biofilm formation by enhancing the antibacterial effect of PLA–PEG films. The fact that imidazole-containing chemical agents target bacterial cell membranes^23^ and inhibit the formation of biofilms^22,23^ and provide the inhibition of microorganisms increases the potential of such films in infection control and treatment strategies. Imidazole-loaded PLA–PEG films have high potential for use in food packaging as well as medical applications. Thanks to its imidazole-related antimicrobial properties, it increases food safety by extending the shelf life of foods. The biodegradability of the PLA–PEG system offers an environmentally friendly packaging opportunity. Food packaging can have its oxygen and moisture permeability be controlled with PLA–PEG films. With the addition of imidazole derivatives, gas barrier properties can be improved, and it may be possible to slow down the oxidative degradation of foods.^24^ In light of all of these studies, the aim of this study was to produce films with methylimidazole and benzimidazole compounds. It was observed that these two compounds did not affect *E. coli* and *S. aureus* bacteria in antibacterial tests. Thereupon, the compounds were interacted with hexachlorocyclotriphosphazene, and methylimidazole substituted cyclotriphosphazene (MCp) and benzimidazole substituted cyclotriphosphazene (BCp) were synthesized. As a result of the antibacterial effect of MCp and BCp compounds, PLA/PEG films were prepared by adding these two compounds at different concentrations. The results showed that the films exhibited good antibacterial properties.

## 2 Materials and Methods

### 2.1 Materials

PLA (PLI005, NaturePlast, melt flow index: 10–30 g/10 min at 190 °C/2.16 kg), poly­(ethylene glycol) (PEG2000, Sigma-Aldrich, 2000 g/mol), tetrahydrofuran (THF, Merck, 99.8%) phosphonitrilic chloride trimer (Sigma–Aldrich, 99%), 2-methylimidazole (Sigma–Aldrich, 99%), benzimidazole (Merck, 98%), *n*-hexane (Merck, 98%), Chloroform (CHCl_3_, CF, J.T. Baker, % 99), dichloromethane (CH_2_Cl_2_, DCM, Carlo-Erba, 99.9%), triethylamine (TEA, Fluka, ≥99.5%), and methanol (CH_3_OH, Sigma-Aldrich, ≥99.8%), agar (Biolab), yeast extract (Biolab), peptone (Biolab), gentamicin sulfate (Merck), tryptic soy broth (Biolab), crystal violet stain (Certistain), ethanol (C_2_H_5_OH, Tekkim, 99.5%) were utilized as purchased without further purification.

### 2.2 Instruments

^1^H NMR spectra were recorded with a Varian INOVA 500 MHz spectrometer in CDCl_3_ at 25 °C. FT-IR spectra were recorded on a PerkinElmer Paragon 1000 spectrometer using the attenuated total reflectance (ATR) method. GPC measurements were conducted on an Agilent GPC Instrument (Model 1100) consisting of a pump, a refractive index detector, and two Waters Styragel columns (HR 5E), using THF as the eluent at a flow rate of 0.3 mL/min at 23 °C and toluene as an internal standard. The average molecular weights and molecular weight distributions of the synthesized polymers were determined with Chem Station for LC (Rev. A. 10.02) software using a calibration curve, developed with linear PS standards known molecular weights. TGA was performed on a TGA/SDTA 851 (Mettler Toledo) thermogravimetric analyzer with a heating rate of 10 °C/min from room temperature to 700 °C under a nitrogen atmosphere. The mechanical tests of obtained films were performed at 10 mm/min crosshead speed with an Instron universal testing machine (Model 3345) at room temperature. The results were reported as an average of at least five parallel measurements. The surface morphology and energy-dispersive X-ray spectroscopy (EDX) analysis of imidazole derivative cyclotriphosphazene-based PLA/PEG composite films were evaluated by SEM (FEI-QUANTA FEG 250-Field Emission scanning electron microscope (FE-SEM)). The acceleration voltage was 30 kV, spot size was 5.0, and the magnifications were 1000x and 2000x. The thickness of the films was determined at different points on each sample surface by using a Mitutoyo digital micrometer. The density of each film (kg/m^3^) was identified by measuring the respective area and thickness of the films. During antibacterial and antibiofilm assays, the optical density (OD_600_) of bacterial cells was measured with a Shimadzu UV-1280 model (Dusiburg, Germany) spectrophotometer, and the density of biofilm layers (OD_575_) was measured with a BMG LABTECH FLUOstar Omega model microplate reader (Offenburg, Germany).

### 2.3 Synthesis of Methyl- and Benzimidazole Cyclotriphosphazenes (MCp and BCp)

The compounds given in Scheme 1 were synthesized according to the literature. Their structural characterizations were performed and compatible results with the literature were obtained.^25,26^

**Scheme 1:**
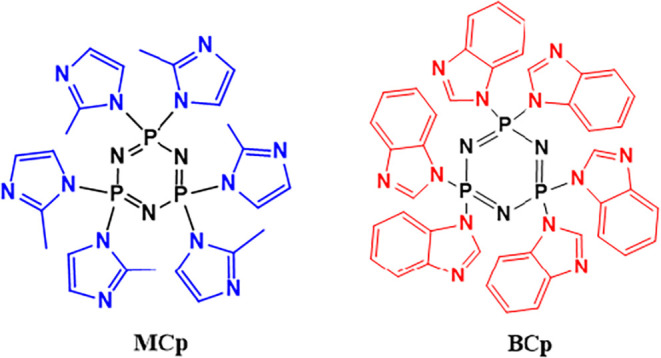
Chemical Formula of MCp and BCp

### 2.4 Preparation of Plasticized PLA-Imidazole-Based Cyclotriphosphazene Films

The prepared methylimidazole cyclotriphosphazenes (MCp) containing PLA/PEG composite films were encoded as MCp0.5, MCp1, MCp3, and MCp5 for 0.5, 1, 3, and 5 wt % methylimidazole content, respectively. Similar coding was done for the prepared PLA/PEG composite films containing benzimidazole cyclotriphosphazenes (BCp). They were coded as BCp0.5, BCp1, BCp3, and BCp5 for 0.5, 1, 3, and 5 wt % benzimidazole content in turn. The PLA, PLA/PEG, PLA/PEG/MCp, and PLA/PEG/BCp composite films were fabricated by a solvent casting method. First, PLA pellets were dried in a vacuum oven at 50 °C overnight and then dissolved completely in chloroform (10 w/v%) using a magnetic stirrer for 2 h at room temperature until a homogeneous and transparent solution was formed. PEG 2000 (10 wt %) was used for plasticizing the PLA pellets. It was shown that the amount of plasticizer has no significant influence on the antibacterial behavior of PLA films; therefore, plasticizer was added at a constant amount. After PEG 2000 was added to the PLA solution dissolved in chloroform, the mixture was stirred for another 1 h at room temperature on a magnetic stirrer. Eight different PLA/PEG/imidazole mixtures were then prepared by adding imidazole derivatives of varying concentrations to this solution. After the imidazole derivatives were added, the mixture was stirred for 3 h at room temperature on a magnetic stirrer. The mixtures were cast into Petri dishes. The chloroform evaporated slowly in the fume hood at room temperature for a period of 24 h and then dried for 2 days in a vacuum oven at 50 °C to remove any residual moisture and solvent. Then, the obtained films were peeled from the Petri dishes carefully and cut into 1 × 1 cm dimensions for antibacterial analysis.

### 2.5 Antibacterial Activity of Imidazole-Based Cyclotriphosphazene Compounds

Gram-negative *E. coli* ATCC 53323 and Gram-positive *S. aureus* ATCC 29213 strains were used to evaluate the antibacterial activity of compounds. Bacterial cells were cultivated in Luria–Bertani broth medium at 37 °C, 180 rpm to obtain young precultured cells. The optical densities of the bacterial suspension were adjusted to 0.1 OD_600_ in LB broth medium. For agar-well diffusion tests, 12% agar (w:v) containing LB medium was prepared and poured into each 9 cm Petri dish at a volume of 17 mL. After polymerization of medium, 100 μL bacterial suspension was inoculated onto LB agar medium and left at room temperature at least 30 min to dry. The wells were perforated by a sterile Pasteur pipet (7 mm diameter). Serial dilutions of each compound were dissolved with chloroform at the concentrations of 1000, 500, 250, 100, 50 μg/mL and 30 μL of each concentration of each compound were transferred into wells. Gentamicin (50 μg/mL) was used as a positive control. The plates were incubated at 37 °C under static condition; after overnight incubation, diameters of inhibition zones were measured (mm).^27^ Diameters of inhibition zones coming from only chloroform application were excluded from the results and given in the related figure.

### 2.6 Antibiofilm Activity of Plasticized PLA-Imidazole-Based Cyclotriphosphazene Films

Gram-negative *E. coli* ATCC 53323 and Gram-positive *S. aureus* ATCC 29213 strains were used to evaluate the antibiofilm activities of PLA/PEG films containing the imidazole derivative cyclotriphosphazene. Bacterial cells were cultivated in Tryptic Soy Broth medium containing 2% glucose (w:v) (TSB_glu_) at 37 °C and 180 rpm to obtain young precultured cells. The optical densities of the bacterial suspension were adjusted to 0.1 OD_600_ in TSB_glu_ medium. Biofilm formation experiments were performed in 6-well cell culture plates containing PLA/PEG films containing the imidazole substituted cyclotriphosphazene (1 × 1 cm^2^). A volume of 3 mL of TSB_glu_ medium containing 0.1 OD_600_ cell suspension was transferred on wells containing the films and incubated at 37 °C under static conditions for 48 h to obtain mature biofilm formation on the films. After incubation, each film was removed from wells and submerged into sterile distilled water for 3 times gently, then transferred onto clean tubes. The films were dyed with 0.05% (w:v) crystal violet stain (3 mL) for 30 min at room temperature. The dyed films were removed from wells and submerged into sterile distilled water for 3 times gently, then transferred onto clean tubes. Ethanol (96% (v:v)) was added onto dyed films and the films were incubated for 10 min at room temperature. Absorbances of samples were measured by a microplate reader at 575 nm wavelength and antibiofilm activity (%) of each application was calculated according to formula given below:^28^

$$biofilm\;\mathrm{in}hibition\;\left(\%\right)=\frac{\mathrm{OD control}-\mathrm{OD}\;s\mathrm{ample}}{\mathrm{OD}\;c\mathrm{ontrol}}\times 100$$

## 3 Results and Discussion

FT-IR analysis results of films obtained by doping imidazole substituted cyclotriphosphazene compounds to PLA/PEG are summarized in Table 1. In the FTIR spectrum of the pure PLA/PEG film, characteristic stretching frequencies for C=O stretching, asymmetric −CH_3_ stretching, symmetric −CH_3_ stretching, and C–O are seen at 1754, 2994, 2941–2877, and 1085 cm^–1^, respectively. C–O–C stretching bands are observed at 1209, 1183, and 1085 cm^–1^. In the FTIR analyses of PLA/PEG/MCp and PLA/PEG/BCp composite films, P=N stretching is expected at 1180–1200 cm^–1^ due to the added material being a cyclophosphazene compound. However, the P=N stretching was detected to be buried within the peaks belonging to PLA due to the small amount of cyclotriphosphazene compound compared to PLA. All of the spectra were similar to PLA/PEG. FT-IR analysis was performed for each concentration of PLA/PEG/MCp and PLA/PEG/BCp films (0.5, 1, 3, and 5 wt %). Since the additive ratio is very low compared to PLA, the bandwidths and intensities of the films did not show any significant change depending on the difference (Table 1).

**Table 1:** FTIR Analysis of the Obtained Films

	υ_(−CH_3_)_ asymmetric stretching	υ_(−CH_3_)_ symmetric stretching	υ_(C=O)_ stretching	υ_(−CH_3_)_ asymmetric bending	υ_(−CH_3_)_ symmetric bending	υ_(C–N)_	υ_(P=N)_	υ_(C–O)_
PLA/PEG	2994	2941, 2877	1754	1454	1359			1183, 1085, 1209
PLA/PEG/MCp0.5	2998	2941, 2885	1750	1453	1359	1270	1182, 1126	1082, 1182, 1126
PLA/PEG/MCp1	2994	2945, 2873	1750	1452	1363	1266	1182, 1128	1082, 1182, 1128
PLA/PEG/MCp3	2998	2941, 2887	1751	1453	1659	1268	1182, 1127	1082, 1182, 1127
PLA/PEG/MCp5	2998	2949, 2886	1752	1453	1360	1275	1182, 1209	1083, 1182, 1209
PLA/PEG/BCp0.5	2994	2945	1750	1452	1355	1268	1182, 1129	1083, 1182, 1129
PLA/PEG/BCp1	2994	2941, 2883	1750	1452	1354	1270	1182, 1129	1083, 1182, 1129
PLA/PEG/BCp3	2999	2941, 2884	1751	1453	1360	1240	1182, 1143	1085, 1182, 1143
PLA/PEG/BCp5	2994	2972, 2945	1750	1452	1363	1268	1182, 1129	1082, 1182, 1129

### 3.1 Mechanical Properties of PLA/PEG/MCp and PLA/PEG/BCp Films

The mechanical test results of PLA/PEG/MCp composites are given in Figure 1. Pure PLA films are naturally brittle, and because of this, they have inadequate mechanical properties for use in industrial applications. It is generally used with plasticizers, and the most common is PEG. PEG is generally used for enhancing the flexibility of PLA as well as its mechanical properties.^8,29–32^ The PLA/PEG film as a control sample in our work has a modulus of 1484 MPa, the addition of MCp until 1 wt % concentration did not affect the modulus, and a decrease was observed after that concentration. This result is compatible with the addition of methenamine to PLA/PEG films as in our previous work.^30^ The incorporation of MCp into PLA/PEG caused a sharp decrease in elongation at break values (%), and it remained constant after a 0.5% concentration. The high elongation at break indicates that the polymer is flexible, soft, and tough like PLA/PEG, but the addition of a filler caused a negative effect. This behavior is attributed to restricted polymer chains caused by increasing filler concentrations.^33^ The yield strength of PLA/PEG is 16.25 MPa. The addition of MCp at 0.5 ratio did not affect the yield strength of PLA/PEG, but after that concentration, an evident decrease was observed until 3% concentration.

**Figure 1:**
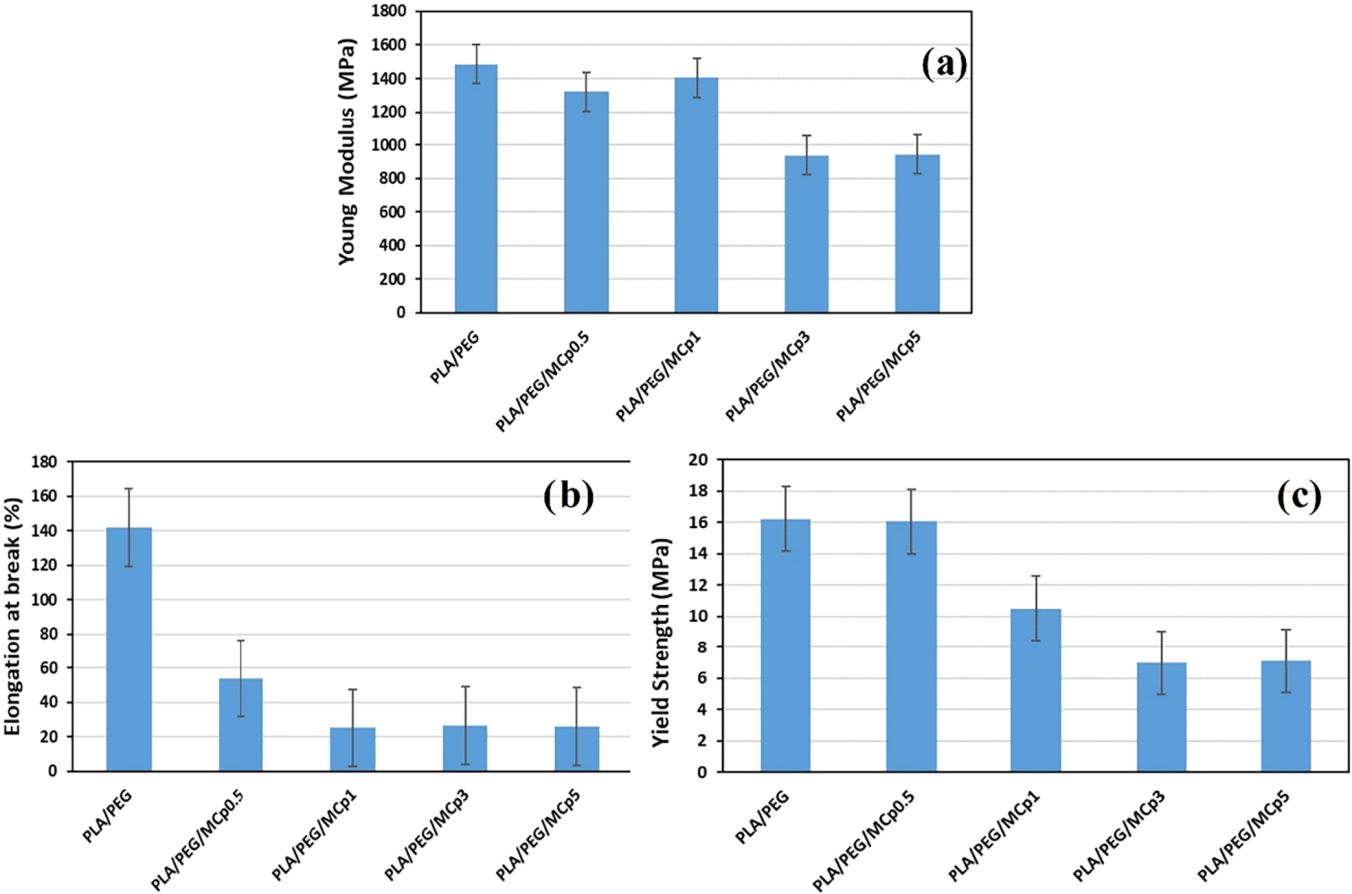
Mechanical properties of PLA/PEG/MCp films at different concentrations: (a) Young modulus, (b) elongation at break (%), and (c) yield strength.

The mechanical test results of PLA/PEG/BCp composites are given in Figure 2. The addition of BCp caused a decrease in modulus values, but there was no significant effect with an increase of concentration. There was observed a serious decrease in elongation at break values similar to MCp addition, which shows that the incorporation of filler into PLA/PEG system did not give the plasticizing effect, and the final films exhibit more brittle behavior when compared to pure to PLA/PEG films. A decrease was also seen at the yield strength in concordance with MCp.

**Figure 2:**
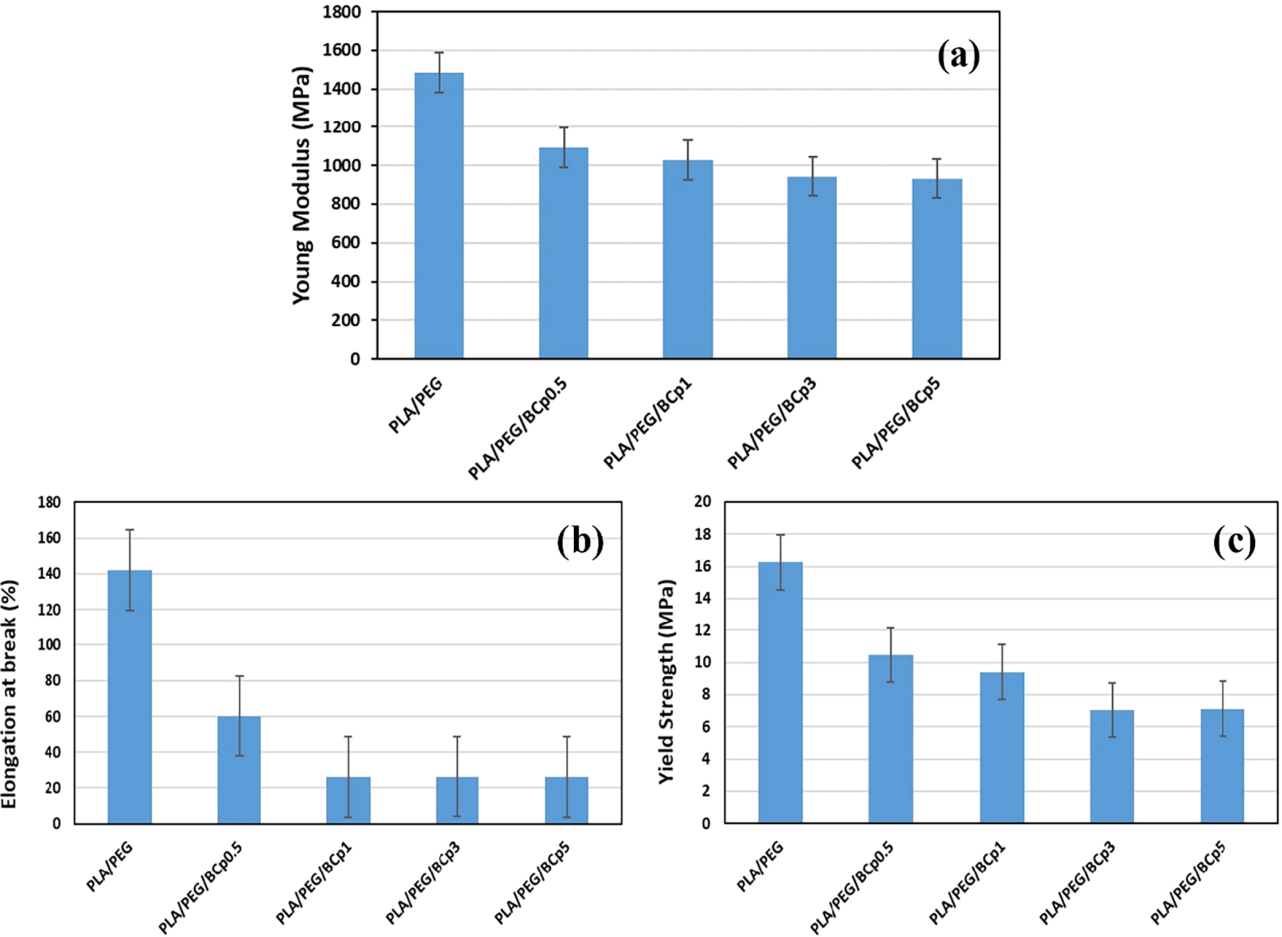
Mechanical properties of PLA/PEG/BCp films at different concentrations: (a) Young modulus, (b) elongation at break (%), and (c) yield strength.

### 3.2 Thermal Properties of PLA/PEG/MCp and PLA/PEG/BCp Composite Films

The effects of BCp and MCp loading on the thermal behavior of PLA/PEG composite films were investigated using DSC and TGA analysis. Numerical values obtained from DSC and TGA analysis are given in Table 2. DSC curves of PLA/PEG composite films are displayed in Figure 3. The glass transition (*T*_g_), cold crystallization (*T*_cc_), and melting (*T*_m_) temperatures of the PLA/PEG composite film were recorded as 45.5 °C, 89.1 °C, and 150.5 °C in turn. When BCp and MCp additives were added to the PLA/PEG polymer, a significant decrease in the *T*_cc_ value was observed. While the *T*_g_ and *T*_m_ values were higher than the *T*_m_ and *T*_g_ values of PLA/PEG at low BCp and MCp concentrations, a general decrease was observed in the *T*_m_ and *T*_g_ values of composites containing BCp and MCp as the concentration increased. Based on these results, the improved thermal properties at low BCp and MCp concentrations can be attributed to these additives acting as nucleating agents. At optimal, lower concentrations, BCp and MCp likely facilitate the formation of more numerous and/or more perfectly ordered crystal structures within the PLA/PEG matrix. This enhanced crystallization results in higher *T*_g_ and *T*_m_ values by creating a more rigid and thermally stable polymer network, as the increased crystallinity requires more energy to disrupt the ordered regions and thereby elevate the glass transition temperature.

**Figure 3:**
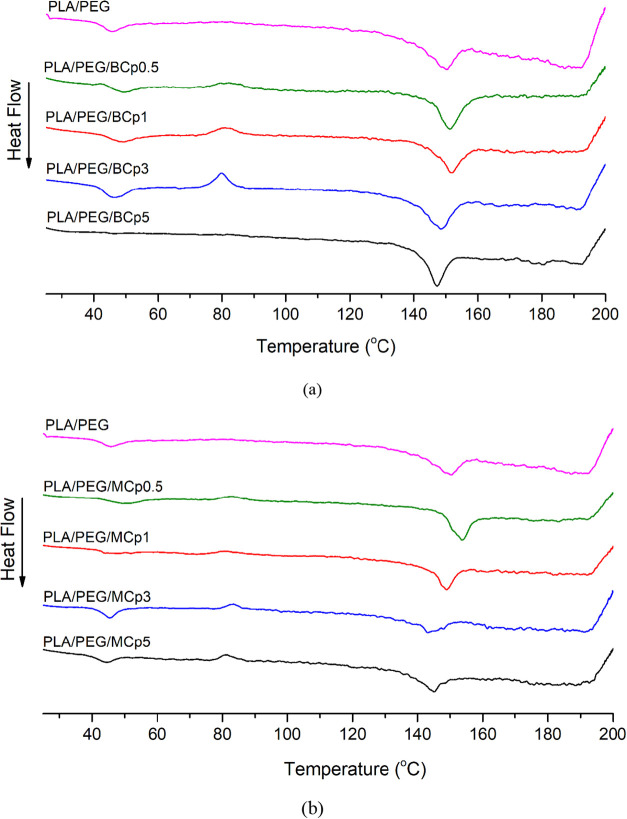
DSC plots of (a) PLA/PEG/BCp and (b) PLA/PEG/MCp composite films.

**Table 2:** DSC and TGA Results of the Composite Films

entry	*T*_g_ (°C)^a^	*T*_cc_ (°C)^b^	*T*_m_ (°C)^c^	*T*_onset_ (°C)^d^	*T*_50_ (°C)^e^	*T*_max_ (°C)^f^	char yield (%)^g^
PLA/PEG	45.5	89.1	150.5	330	353	378	2.9
PLA/PEG/MCp0.5	50.3	82.4	153.8	345	364	384	1.2
PLA/PEG/MCp1	46.4	81.5	149.0	344	364	383	1.2
PLA/PEG/MCp3	45.5	83.2	143.2	334	356	380	1.1
PLA/PEG/MCp5	44.6	81.2	145.4	340	361	384	2.1
PLA/PEG/BCp0.5	49.5	81.3	151.2	338	358	376	1.4
PLA/PEG/BCp1	48.6	80.9	151.8	335	354	374	1.0
PLA/PEG/BCp3	46.6	79.9	148.5	336	358	379	2.0
PLA/PEG/BCp5	45.9		147.1	332	358	385	2.5

a *T*_g_ denote the glass transition of the films in the first heating run of the DSC experiments in turn.

b *T*_cc_ denote the cold crystallization of the films in the first heating run of the DSC experiments in turn.

c *T*_m_ denote the melting point temperature of the films in the first heating run of the DSC experiments in turn.

d *T*_onset_ represents the onset decomposition temperature of the films.

e *T*_50_ represents the temperatures of weight loses at 5% and 50% in turn.

f *T*_max_ is the temperature that corresponds to the maximum rate of weight loss.

g The percentage weight remaining at 700 °C.

The percent weight–temperature curves of the composite films are displayed in Figure 4, and the data related to their initial decomposition temperature (*T*_onset_), decomposition temperatures at 50 wt % (*T*_50_), maximum thermal decomposition temperature (*T*_max_), and char yield are reported in Table 2. The addition of BCp and MCp compounds did not have a negative effect on the thermal stability of the PLA/PEG film. The thermal stability of PLA/PEG films obtained by adding imidazole derivative cyclotriphosphazene compounds as additives increased slightly, depending on the added concentration. While the temperature (*T*_onset_) at which the pure PLA/PEG film started to decompose was 330 °C, this temperature increased to 332–345 °C with the addition of additives. In general, the same effect was detected at the *T*_max_ and *T*_50_ temperatures. Based on these results, the improved thermal stability of the PLA/PEG composite films with the addition of BCp and MCp compounds can be attributed to their fire-retardant or char-forming capabilities. These additives likely promote the formation of a protective char layer at elevated temperatures, which insulates the underlying polymer from further degradation, thereby increasing the onset of decomposition (*T*_onset_), the temperature at 50% weight loss (*T*_50_), and the maximum decomposition temperature (*T*_max_). This suggests that the imidazole derivative cyclotriphosphazene compounds can act as thermal barriers or radical scavengers, stabilizing the PLA/PEG matrix against thermal degradation.

**Figure 4:**
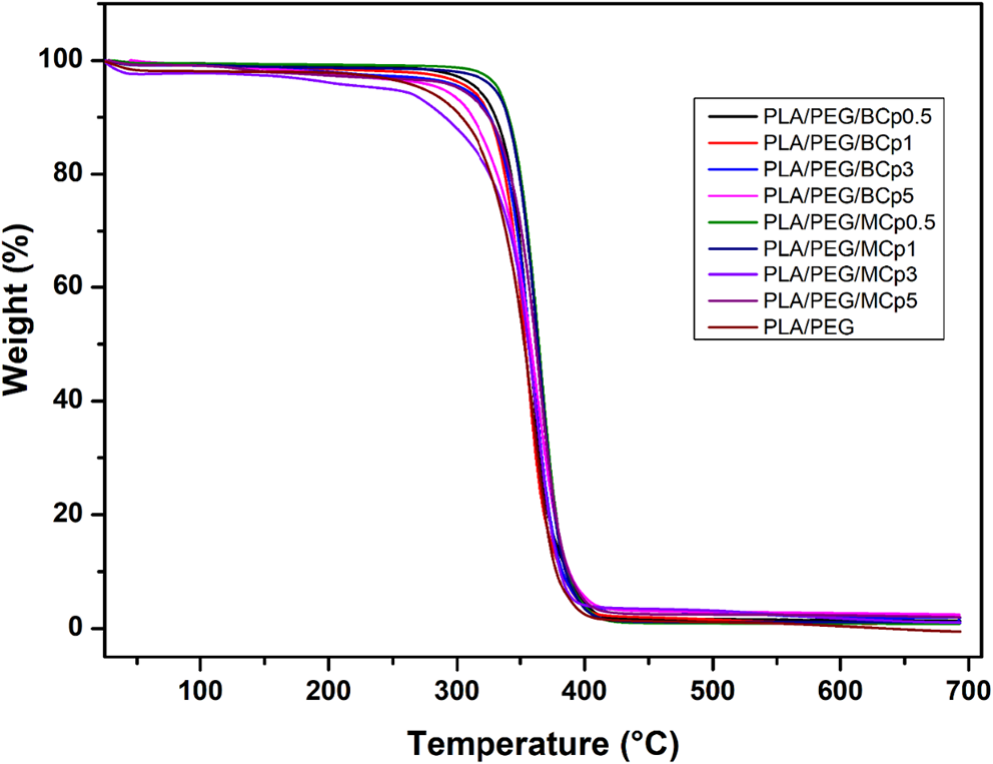
TGA plots of the composite films.

### 3.3 Surface Morphology of PLA/PEG/MCp and PLA/PEG/BCp Films

SEM micrographs of pure PLA/PEG and PLA/PEG/MCp and PLA/PEG/BCp films are shown at 500x and 2000× magnifications in Figure 5. When the SEM micrographs are examined, the PLA/PEG surface exhibited a smooth, homogeneous, and flat surface with good dispersion of PEG in the PLA matrix in terms of morphology.^31^ The changed morphology of the obtained films and the observed clusters are attributed to the MCp and BCp additive compounds. The distributions of the clusters are relatively uniform, as can be seen from the data in Figure 5. It can be said that the porous structure in pure PLA/PEG films comes from PEG. However, it was observed that the number of pores decreased as the amount of MCp and BCp increased. In addition, larger and different pore sizes were obtained as the amount of additives increased. It was observed that agglomeration on the PLA/PEG film surface containing MCp and BCp increased at the highest additive concentrations (PLA/PEG/MCp5 and PLA/PEG/BCp5).

**Figure 5:**
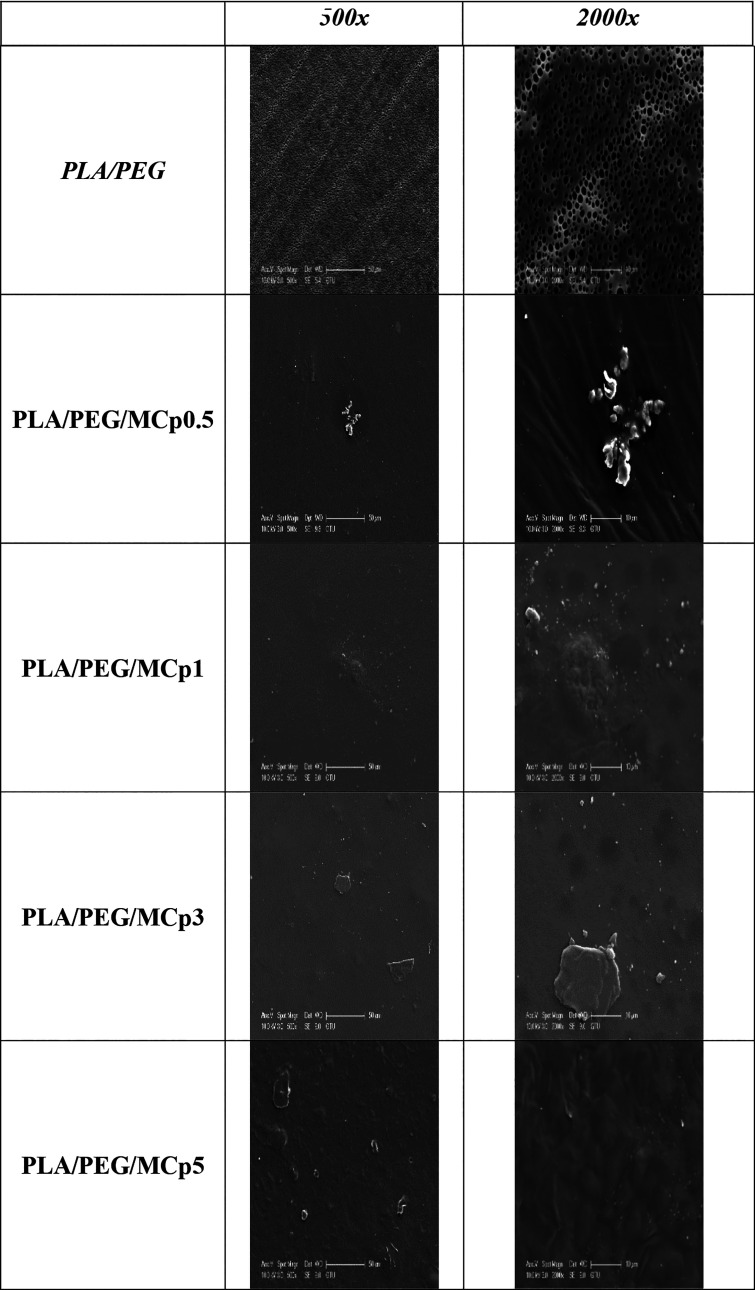

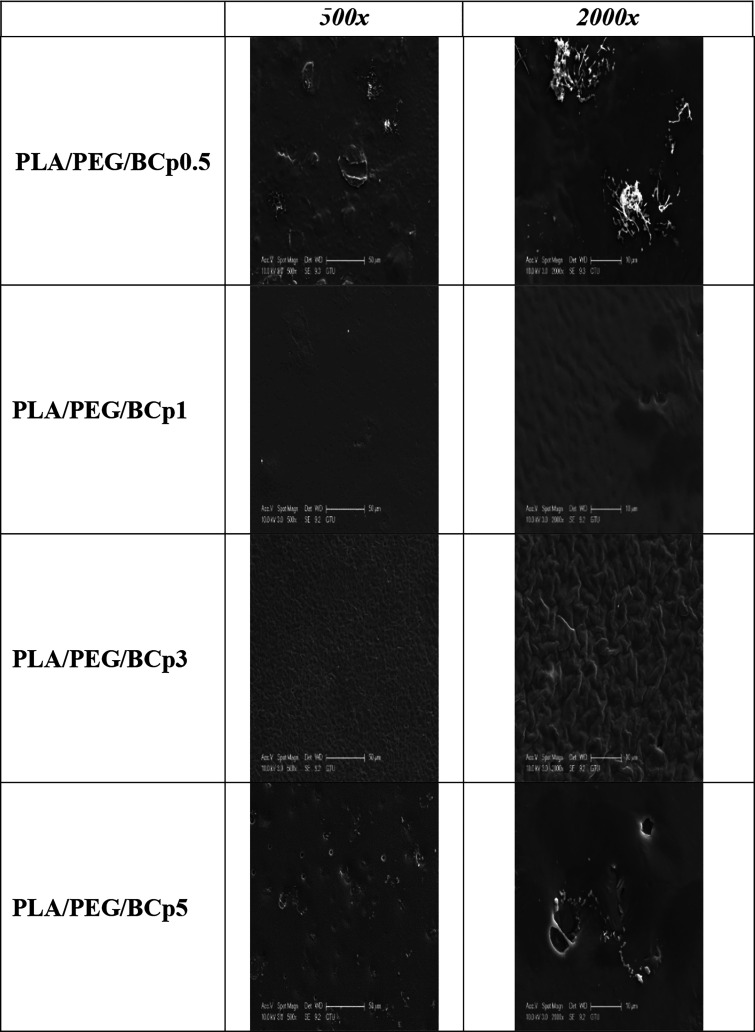
SEM images of PLA/PEG and prepared composites films.

In the EDX analysis of PLA/PEG, traces of elements belonging to C and O atoms are observed (Figure 6a). When the EDX spectra of the added films are examined, the films with the lowest cyclophosphazene addition (PLA/PEG/MCp0.5 and PLA/PEG/BCp0.5) were selected, and traces of P and N elements coming from the cyclophosphazene compounds added to the PLA/PEG content are also evident (Figure 6b,c). EDX analysis was applied to films at all concentrations, and traces of P and N elements belonging to the cyclophosphazene additive compounds were detected in all of them.

**Figure 6:**
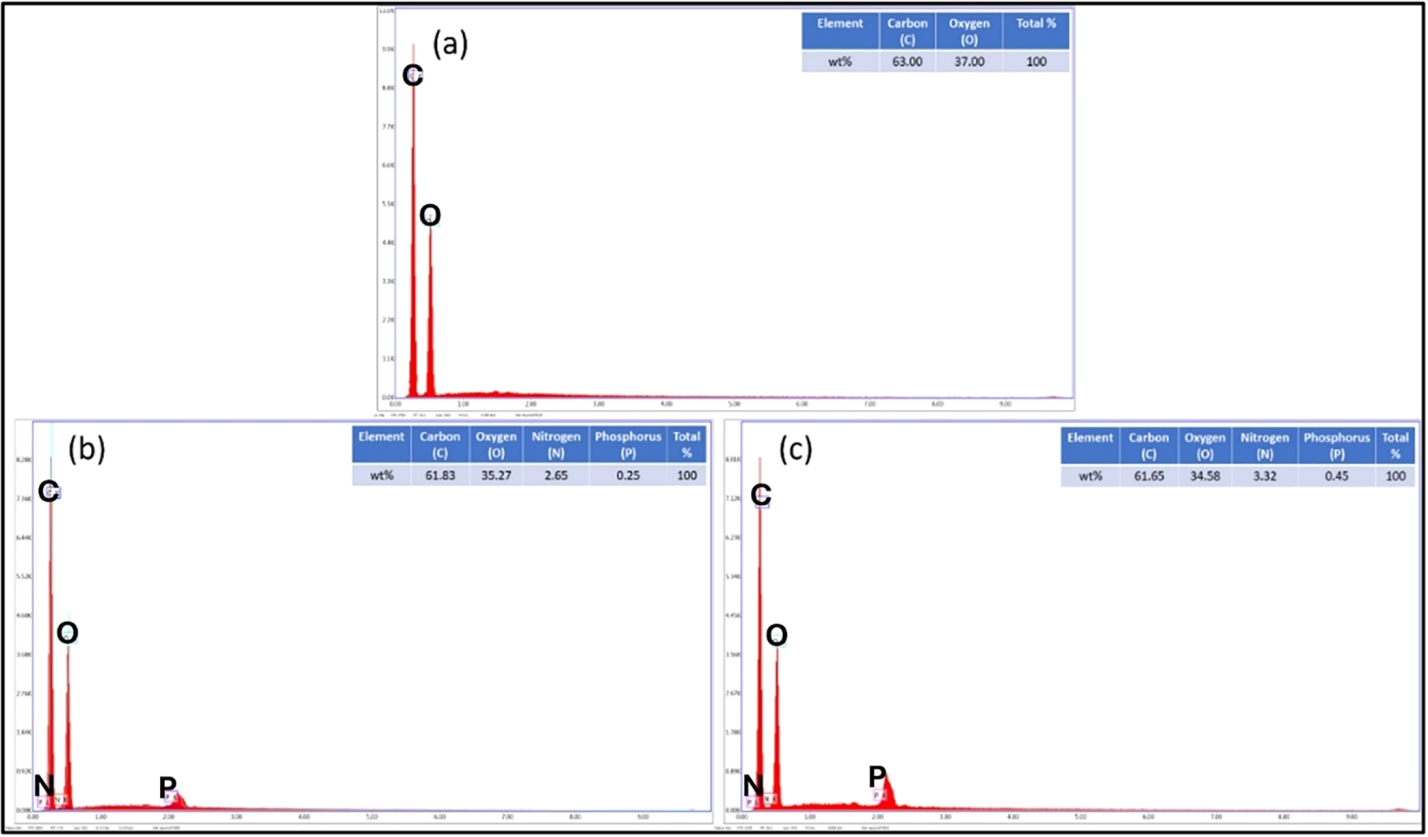
EDX spectra of (a) PLA/PEG, (b) PLA/PEG/MCp3, and (c) PLA/PEG/BCp3, as well as EDX maps displaying the N atom and the atomic composition of the spectrum.

### 3.4 Antibacterial Assessment of MCp and BCp Compounds

Antibacterial activities of MCp and BCp compounds at various concentrations were tested against *E. coli* ATCC 53323 and *S. aureus* ATCC 29213 strains via the agar-well diffusion technique by measuring the inhibition zone diameter after incubation. MCp and BCp compounds treatment caused bacterial inhibition on both strains in a dose-dependent manner. MCp compound was more effective than BCp at all concentrations against *E. coli* ATCC 53323 (12-1 mm and 5-2 mm, respectively) and *S. aureus* ATCC 29213 strains (13-1 mm and 5-2 mm, respectively). Inhibition zones caused by MCp application (1000 μg/mL) were measured as 12 and 13 mm for *E. coli* ATCC 53323 and *S. aureus* ATCC 29213, respectively. After BCp application (1000 μg/mL), inhibition zones were measured as 5 mm for both *E. coli* ATCC 53323 and *S. aureus* ATCC 29213 (Figures 7 and 8).

**Figure 7:**
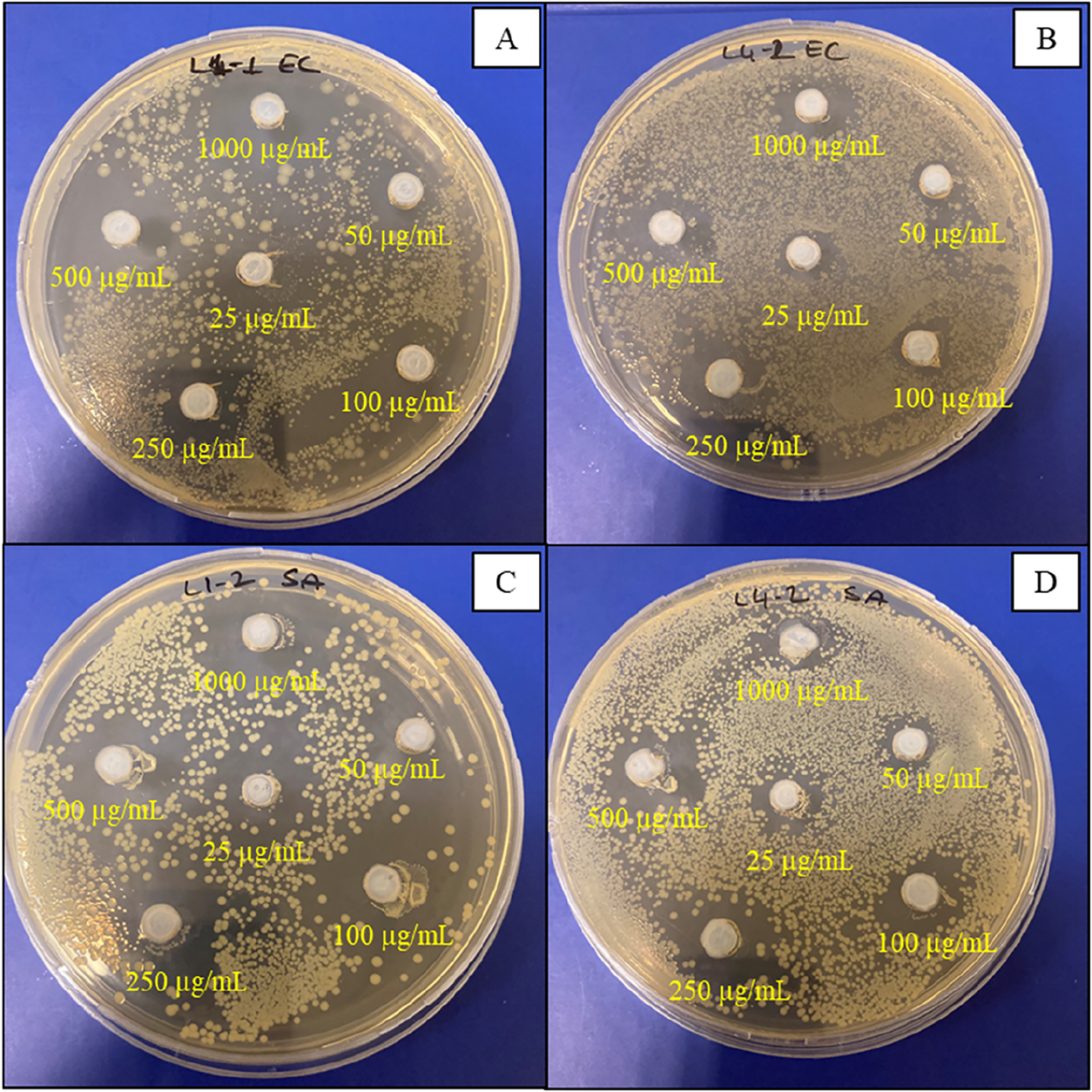
Screening of antibacterial activity of MCp and BCp compounds against *E. coli* ATCC 53323 and *S. aureus* ATCC 29213 strains. (A) *E. coli* ATCC 53323-MCp, (B) *E. coli* ATCC 53323-BCp, (C) *S. aureus* ATCC 29213-MCp, and (D) *S. aureus* ATCC 29213-BCp.

**Figure 8:**
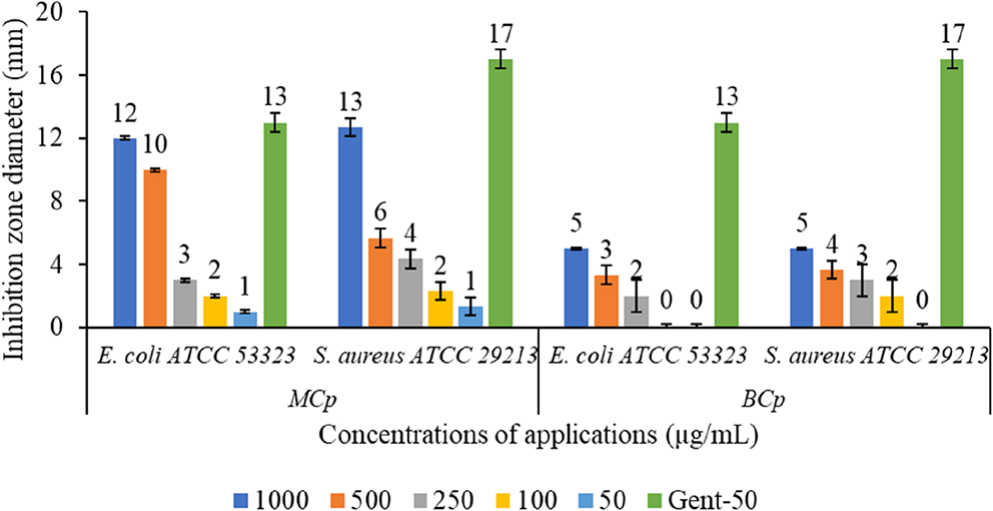
Antibacterial activity results of MCp and BCp compounds against *E. coli* ATCC 53323 and *S. aureus* ATCC 29213 strains. Diameters of the wells (7 mm) were excluded from the total diameter zones of inhibition (mm). Values are the mean of triplicate measurements; error bars represent standard deviations.

### 3.5 Antibiofilm Assessment of PLA/PEG/MCp and PLA/PEG/BCp Films

*E. coli* ATCC 53323 and *S. aureus* ATCC 29213 were used to compare the antibiofilm activities of PLA/PEG/MCp and PLA/PEG/BCp films. It was identified that PLA/PEG/MCp films had antibiofilm activity against mature *S. aureus* biofilm (58–66%), but not against mature *E. coli* biofilm (0%). The most effective application of PLA/PEG/MCp films was determined as MCp-0.5 with an inhibition rate of 66% against *S. aureus* biofilms. Against both mature biofilm of *E. coli* ATCC and *S. aureus* strains, PLA/PEG/BCp films had biofilm inhibitory activity at the rate of 13–80% and 42–63%, respectively. The most effective application of PLA/PEG/MCp films was BCp1 with an inhibition rate of 63% against *S. aureus* biofilm formation (Figure 9). Compared with MCp, BCp showed higher inhibitory activity on biofilm formation by affecting Gram-negative and -positive test bacteria. In another study, antibacterial, antibiofilm, and anticancer activities of fucoidan-loaded zeolitic imidazole framework (FU@ZIF-L) nanocomposites were investigated. FU@ZIF-L showed potent antibacterial and biofilm disrupting effects on *S. aureus* and *E. coli*. Especially at a concentration of 100 μg/mL, it inhibited *S. aureus* biofilm formation by 70% and *E. coli* biofilm formation by 81%. It was reported that the antibacterial effect was mediated by Zn^2+^ ions disrupting the bacterial cell membrane and fucoidan increasing the production of ROS (reactive oxygen species).^34^

**Figure 9:**
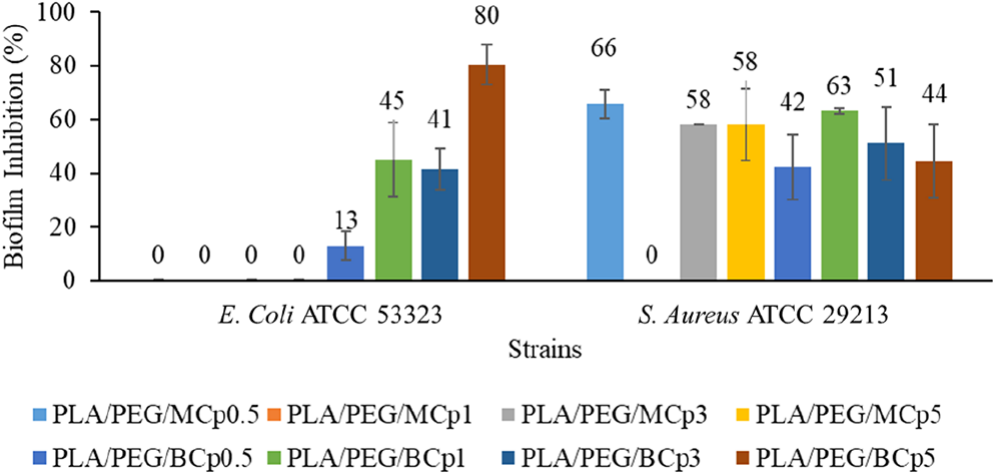
Antibiofilm activity results of PLA, PLA/PEG, PLA/PEG/MCp, and PLA/PEG/BCp composite films against *E. coli* ATCC 53323 (EC) and *S. aureus* ATCC 29213 (SA). Values are mean of triplicate measurements; error bars represent standard deviations.

## 4 Conclusions

In this study, imidazole substituted cyclotriphosphazene compounds (MCp and BCp), which are expected to have antimicrobial properties, were synthesized according to the literature as additive materials and their structural characterizations were performed. These compounds were mixed with PLA/PEG solution at different concentrations (0.5, 1, 3, 5 wt %), and PLA/PEG/MCp and PLA/PEG/BCp films were prepared by a simple solvent casting method. The thermal, mechanical, and biological activities of both the pure additive MCp and BCp compounds and the PLA/PEG films prepared by adding these compounds were comparatively examined. When the thermal stability of the films was examined, it was found that the temperatures at which the PLA/PEG films started to decompose increased slightly, although not significantly, with the addition of BCp and MCp. As a result of mechanical tests, it was observed that the additive compounds negatively affected the mechanical properties of the PLA/PEG films. When compared to the pure PLA/PEG film, there were decreases in the yield strength, Young’s modulus, and elongation at break of the films after the addition of MCp and BCp compounds.

The antibacterial and antibiofilm activities of MCp and BCp compounds were evaluated against *E. coli* and *S. aureus* strains. The results demonstrated that both compounds exhibited dose-dependent antibacterial effects, with MCp showing superior inhibition zones compared to those of BCp on both bacterial strains. Notably, MCp displayed the highest inhibition zone diameters at 1000 μg/mL, measuring 12 and 13 mm against *E. coli* and *S. aureus*, respectively. Additionally, the PLA/PEG/MCp and PLA/PEG/BCp films were tested for antibiofilm activity, where PLA/PEG/MCp films were particularly effective against mature *S. aureus* biofilms with a maximum inhibition rate of 66%, while PLA/PEG/BCp films exhibited broader biofilm inhibitory effects against both Gram-positive and Gram-negative bacteria. The higher antibiofilm activity of BCp suggests its potential in preventing bacterial biofilm formation.

These findings indicate that the newly synthesized MCp and BCp compounds along with their film formulations could be promising candidates for the development of antibacterial and antibiofilm materials, offering new strategies to combat biofilm-associated infections. In light of the successful results obtained from antimicrobial experiments in this study, it was observed that the MCp compound had a serious antibacterial effect on Gram-positive and Gram-negative bacteria, and this effect was almost on a similar scale to the control drug Gent-50. Also, it is thought that PLA/PEG/BCp5 films can find application areas especially in antibacterial packaging and biomedical fields, for example, wound dressing, thus competing with traditional petroleum-based polymeric materials.
